# NO signalling in synaptic transmission

**DOI:** 10.1186/1471-2210-11-S1-O21

**Published:** 2011-08-01

**Authors:** Doris Koesling, Angela Neitz, Thomas Mittmann, Evanthia Mergia

**Affiliations:** 1 Department of Pharmacology, Medical School, Ruhr-University Bochum, Germany; 2 Institute of Physiology & Pathophysiology, Med.-Center of the Johannes-Gutenberg-University Mainz, Germany

## Background

The NO/cGMP signaling cascade has been proposed to play a role in long-term potentiation (LTP) and the modulation of synaptic transmission. Nitric oxide is formed enzymatically by NO synthases (NOS); two NOS, the endothelial and neuronal isoform (eNOS, nNOS) produce NO as a signalling molecule. Functionally, NO has been reported to act as a retrograde messenger that is generated postsynaptically to increase the neurotransmitter release presynaptically. The NO effects are mediated by the NO-sensitive guanylyl cyclases (NO-GC), which by the formation of cGMP transduce the NO signal. Two functionally indistinguishable isoforms of the NO-GCs, NO-GC1 and NO-GC2 exist.

## Results

To elucidate their physiological role, we generated KO mice in which either one of the isoforms is deleted. Surprisingly, LTP measured in hippocampus and visual cortex was abolished in both KO strains indicating that both NO receptors are required for the induction of LTP.

Measurements of the NO-GC isoform content in hippocampus revealed expression of both isoforms in glutamatergic and GABA-ergic neurons. Glutamatergic synaptic transmission was studied in the hippocampal CA1 region of the NO-GC1 KO, NO-GC2 KO and WT mice in whole-cell recordings. Analysis of the presynaptic glutamate release revealed a reduction of the neurotransmitter release in the NO-GC1 KO under basal and stimulated conditions whereas analysis of postsynaptic properties revealed a reduction of NMDA receptor currents in the NO-GC2 KOs.

## Conclusion

Our results suggest that the NO/cGMP signaling cascade enhances the strength of glutamatergic synaptic transmission on both sides of the synaptic cleft.

